# Research and progress of focused ultrasound in the treatment of Alzheimer’s disease

**DOI:** 10.3389/fneur.2023.1323386

**Published:** 2023-12-21

**Authors:** Xishun Ma, Tongxia Li, Lizhen Du, Tongliang Han

**Affiliations:** ^1^Department of Ultrasound, Qingdao Hospital, University of Health and Rehabilitation Sciences (Qingdao Municipal Hospital), Qingdao, China; ^2^Department of Tuberculosis, Qingdao Chest Hospital, Qingdao, China

**Keywords:** Alzheimer’s disease, neurodegenerative disease, nervous system, focused ultrasound, therapy

## Abstract

Alzheimer’s disease is one of the most common degenerative diseases of the central nervous system, with progressive cognitive and memory impairment and decreased ability of daily life as the cardinal symptoms, influencing the life quality of patients severely. There are currently approximately 46 million people living with Alzheimer’s disease worldwide, and the number is expected to triple by 2050, which will pose a huge challenge for healthcare. At present, the Food and Drug Administration of the United States has approved five main drugs for the clinical treatment of Alzheimer’s disease, which are cholinesterase inhibitors tacrine, galantamine, capalatine and donepezil, and N-methyl-d-aspartate receptor antagonist memantine, although these drugs have shown good efficacy in clinical trials, the actual clinical effect is less effective due to the existence of blood brain barrier. With the continuous development of ultrasound technology in recent years, focused ultrasound, as a non-invasive treatment technique, may target ultrasound energy to the deep brain for treatment without damaging the surrounding tissue. For the past few years, some studies could use focused ultrasound combined with microvesicles to induce blood brain barrier opening and targeted drug delivery to treat Alzheimer’s disease, providing new opportunities for the treatment of Alzheimer’s disease. This article reviews the application research and progress of focused ultrasound in the treatment of Alzheimer’s disease, in order to provide new directions and ideas for the treatment of Alzheimer’s disease.

## 1 Introduction

Alzheimer’s disease (AD) is a chronic degenerative disease of the central nervous s ystem, and its pathological mechanism is relatively complex, involving in neurochemistry, cerebrovascular metabolism and inflammatory pathways changes (1). At present, the recognized pathological mechanism is the amyloid cascade theory, which is characterized by senile plaques caused by the deposition of β-amyloid protein (Aβ) and neuronal fibrillary tangles within nerve cells (2). Deposition of Aβ polymers can induce synaptic decline and inflammatory response, as well as axonal degeneration through microtubule-associated tau protein hyperphosphorylation (3). In addition, AD pathology can also show neurofibrillary tangles and choline neuron degeneration. The former is composed of hyperphosphorylated tau protein, and its hyperphosphorylation is triggered by the deposition of Aβ protein, which ultimately leads to neuronal genetic modification and degeneration and brain atrophy (4). The latter choline neuron dysfunction is also related to Aβ protein plaque load, which can eventually lead to cognitive dysfunction (5). Therefore, preventing the formation of Aβ amyloid plaques or accelerating their clearance rate is the key to treating AD.

FUS was once limited in its use in the brain because the skull blocked the ultrasound. With the development and optimization of phased array transducer, by making helmet type treatment head, the transducer array element can be tightly fitted to the skull so that the focus can be precisely located in the brain, and the temperature measurement of module can ensure that the temperature of the skull is within a safe range, making focused ultrasound treatment of brain diseases a reality (6).

Unlike thermal ablation of conventional focused ultrasound, low-intensity focused ultrasound (LFUS) combined with intravenously injected microvesicles can produce local cavitation and open blood brain barrier (BBB) in a few seconds. Due to the low ultrasonic energy used in this process, there is almost no thermal effect, which greatly reduces the damage of ultrasound to brain tissue, skull and blood vessels (7).

LFUS is conducting preclinical and clinical studies in the areas of tissue thermal ablation, neuromodulation, and BBB opening (8). Compared with conventional diagnostic ultrasound, the frequency used for intracranial ultrasound therapy is 650 kHz or 220 kHz, and in recent years, LFUS frequencies below 3 MHz have also shown therapeutic potential for neurological diseases, including AD (9, 10). Studies have shown that 2 MHz LFUS can reversibly block peripheral nerve conduction and has a neuroregulatory effect, which is expected to be applied to the consciousness disorders and screening of people with thermal ablation treatment (5). The main effects of LFUS with different frequency and intensity are different, including neuromodulation, cavitation effect, activation of mechanically sensitive ion channels, pore formation, thermal effect, etc (11).

In 2018, Nir L et al. successfully conducted a clinical trial of 5 AD patients in stage I with using focused ultrasound to open BBB, proving that focused ultrasound can safely, reversibly and repeatedly open BBB in AD patients (12), suggesting that ultrasonic focusing technology can be used in the treatment of AD in non-invasive or adjuvant therapy way.

## 2 Improvement of LFUS alone in AD

Studies have shown that LFUS treatment alone can reduce Aβ and tau lesions in AD animal models, and promote hippocampal neurogenesis, increase nerve growth factor content, activation of TRKA-related survival signaling pathways, and improvement of cognitive function (13, 14).

One study found that LFUS (1 MHz) alone reduced phosphorylated tau burden and improved spatial learning and motor deficits in K369I tau transgenic mouse models (15). A study using 5XFAD mice showed that whole brain application of LFUS (1.875 MHz) alone for 3 weeks significantly improved cognitive impairment associated with cerebral blood flow and reduced IBA-1-positive microglia and amyloid plaques (10). Notably, results using APP23 mouse models showed that LFUS (1 MHz, 0.7 MPa) combined with microvesicles reduced amyloid plaques in mouse brain spaces and improved memory function, whereas LFUS alone was not sufficient to open the BBB and clear amyloid plaques in mouse brain Spaces.

In conclusion, there are few preclinical studies on the effects of LFUS alone in the absence of microvesicles, and more studies are needed to demonstrate the efficacy and safety of LFUS alone.

## 3 Improvement of LFUS combined with microbubbles in AD

In 2008, Raymond et al. (16) first used focused ultrasound combined with microbubbles to open BBB to assist drug delivery, which could deliver mouse IgG antibodies to the brain of AD mice. In this study, two transgenic AD mice APPs we were used: PSEN1dE9 and PDAPP evaluated the effect of ultrasound combined with microbubble on IgG antibody delivery in mice at a wide range of weeks of age (9–26 months of age). The results confirmed that ultrasound combined with microbubble irradiation can effectively deliver antibodies in unilateral brain region, and the delivery capacity was consistent in mice of different weeks of age and different transgenic mouse models. Its good repeatability and stability have been proved.

Jordão et al. (17) found that focused ultrasound treatment could also improve the clinical symptoms of AD model mice without exogenous antibodies. In this study, only by using FUS combined with microbubble therapy, after 4 days of treatment, it was found that the load of Aβ protein plaques in the brain irradiated by FUS was significantly reduced. The pathological results suggested that the reason may be that FUS irradiation promoted the transport of endogenous antibodies such as IgG and IgM and bound to Aβ protein plaques. In addition, the activation of astrocytes and microglia after FUS irradiation further promoted the clearance of Aβ protein plaques.

LFUS can make the microvesicle contrast agent produce stable concussion, and instantly open the BBB by separating the tight connections of endothelial cells, thus activating the innate immune system to clear amyloid plaques and promote neurogenesis (18, 19). Advances in skull distortion correction techniques and phased array sensors have led to the development of fully noninvasive therapeutic LFUS, combined with magnetic resonance guidance for positioning, real-time monitoring, and thermal feedback, which could produce therapeutic effects at any selected site from the cerebral cortex to deep structures (20). Magnetic resonance guided LFUS combined with intravenous microvesicles has been established as a safe, repeatable, and targeted way to open the BBB (21). This phenomenon is thought to be caused by ultrasonic cavitation, and the results are oscillations of microvesicles and a reversible breakdown of the tight connections between endothelial cells that mechanical forces in the capillary wall induce (20, 22).

Studies have shown that in the absence of any drug delivery, LFUS combined with microbubble contrast agents could cause Aβ to move from brain parenchyma to cerebrospinal fluid and then from lymphatic vessels to excreting brain tissue, and increased uptake of Aβ by microglia, moreover, improved behavioral disorders and memory deficits in animal models and delayed the disease process (15, 23, 24). After multiple LFUS (0.996 MHz, 0.64 MPa) treatmenst, the learning and memory ability of 3xTg-AD mice were significantly improved, and the deposition of Aβ and phosphorylation of tau protein in the brain region treated by LFUS were improved, and axons were increased. Confocal microscopy showed that activated microglia engulfed Aβ (25). The results also suggested that different ultrasound parameters or animal models of AD might have different effects on cells.

## 4 Improvement of LFUS combined with drugs in AD

The researchers found that LFUS (1.68 MHz) combined with intravenous gamma globulin (IVIg) delivered IVIg to the hippocampus of TgCRN8 mice 39 times more efficiently than without LFUS, and quadrupled hippocampal neurogenesis (9). When the researchers used tau protein 2 N fragment specific antibody RN2N combined with ultrasound (1 MHz, 0.7 MPa), it was found that the efficiency of RN2N entering the brain of PR5 mice and being taken up by neurons was significantly increased, and the efficacy was significantly enhanced (14). It could be seen that ultrasound was a feasible method to enhance the efficiency of biologics crossing the BBB and enhanced the curative effect.

## 5 Improvement of magnetic resonance guided focused ultrasound in AD

In 2011, Time magazine named magnetic resonance guided focused ultrsound (MRgFUS) technology as one of the world’s top 50 inventions and has caused extensive research around the world.

A Phase II clinical trial (NCT03671889) using an ultrasound helmet (ExAblate 4,000 Low-frequency System Type 2 helmet sensor, 220KHz) in three women with AD showed that MR-guided LFUS could reversibly open the BBB and deliver microvesicles to the hippocampus. It did not cause infarction, edema, demyelination, bleeding or gliosis, and the patient’s neurological and psychological examination results remained stable after the completion of the experiment (20). MRgFUS combined with microvesicles resulted in spatially precise transient BBB opening within the hippocampus and entorhinal cortex in AD patients (20, 26). At the same time, a Phase I clinical trial (NCT02986932) demonstrated that MRgFUS might temporarily affect neurological function, but would recover within a day. It suggested that LFUS was very safe for the treatment of human central nervous system diseases. In addition, the size of the BBB opening could be controlled by changing the focused ultrasound parameters, making the technique more accurate and controllable.

Jordao JF et al. confirmed that MRgFUS could deliver anti-Aβ antibody by opening BBB for the first time, and injected gadolinium contrast agent, microbubble and anti-Aβ antibody BAM 10 into TgCRND8 model mice (27). MRgFUS was used to irradiate four regions in the right hippocampus of the brain of mice. It was found that FUS can effectively promote the contrast agent and BAM 10 to enter the cortex. Four days after treatment, the number and area of Aβ protein plaques in the irradiated brain region were significantly reduced compared with the unirradiated control group, and the concentration of antibodies required was lower, as low as 40 μg, which was 100 times less than the amount of antibodies used in Raymond’s study (16), demonstrating that FUS could not only deliver antibodies efficiently, but also improve the pathology of AD in a short time.

In the later stage, MRgFUS was used to irradiate the hippocampal region of TgCRND8 model mice once a week. In the absence of foreign antibodies, AD model mice treated with FUS combined with microbubble showed improved spatial memory and behavioral symptoms, and such improvement might be related to the reduction of Aβ protein plaques (28). Because the above studies used different antibodies, the duration of their efficacy was unknown. Poon et al. (29) further studied the time process of BBB opening by focused ultrasound in the AD mouse model, and found that without the use of foreign antibodies, a single FUS irradiation could reduce Aβ protein plaques by about 60% within 2 days, and the effect could last for 2 weeks. Following the treatment with three to five fortnightly FUS treatments, the treated group was found to have 27% fewer Aβ protein plaques and 40% fewer plaques than the untreated group, which indicated that biweekly irradiation could be a potentially effective treatment.

Leinenga et al. (30) found in the experiment of opening BBB of 10 APP23 transgenic mice that compared with 10 mice treated with sham, the number and surface area of Aβ plaques in mice treated with MRgFUS were reduced, and the mice performed better in three memory tasks: Y-maze, novel object recognition and active avoidance experiment. In addition to reducing Aβ plaques, ultrasonic treatment can also increase the occurrence of hippocampal neurons in AD mouse models, and induce the phospholipids inositol 3 kinase-protein kinase-rapamycin target protein (PI3K-Akt–mTOR) signaling pathway. Activation of the PI3K-Akt–mTOR pathway can improve the survival of newborn neurons in the dentate gyrus in the AD mouse model, and enable newborn neurons to avoid dendritic growth defects caused by Aβ (31). Jalali et al. (32) found that the opening of BBB mediated by ultrasound combined with microvesicle contrast agent could induce the activation of PI3K-Akt signaling pathway in neurons of AD rats, and these animal experimental results provided reliable theoretical support for clinical studies in AD patients. At present, clinical trials evaluating the safety and feasibility of MRgFUS have been applied to patients with early AD (33). Lipsman et al. (12) included 5 patients with AD and aimed to explore whether MRgFUS could safely open BBB in patients with mild to moderate Aβ-positive AD. The study results showed that MRgFUS could safely and reversibly open BBB without other adverse events.

## 6 Improvement of scanning focused ultrasound in AD

The study found that the use of scanning ultrasound (SUS) might be another viable treatment for AD. In the same condition independent of antibodies, whole brain focused ultrasound irradiation of transgenic mice once a week for 7 weeks showed activation of microglia, Aβ protein plaque was reduced by 75%, and the hippocampal-dependent spatial learning and memory ability was rebuilt (30). Significantly, the team further irradiated the whole brain of P301L mutated tau transgenic mice with anti-tau antibody combined with FUS at a later stage, and found that FUS could promote the transport of tau single chain antibodies to nerve cells, the phosphorylation level of tau protein at different sites was reduced, and the anxiety behavior of mice was significantly improved. Since the antibodies used in this study were single-chain antibodies and antibodies targeting tau protein, it provided a new treatment for AD that was different from targeting Aβ protein.

### 6.1 Prospect and challenge

With the continuous development of ultrasound technology, FUS can induce BBB opening in a non-invasive, reversible and targeted manner, which has a broad application prospect in the treatment of brain diseases. A large number of pre-clinical studies have shown that FUS induction of BBB opening and drug delivery can alleviate the pathological features of AD, stimulate hippocampal neurogenesis and improve memory function to a certain extent, which is expected to be a potential treatment for AD. However, there are still many problems in practical clinical application. Although FUS breaks through the limitations of BBB to achieve innovation in drug delivery mode, there is a lack of studies on the pharmacokinetics and drug dose selection of this central drug delivery mode. How the parameters and size of BBB opened by focused ultrasound affect the therapeutic effect of drugs has not been studied, and a large number of experiments are needed to confirm it. In addition, the safety of BBB opening by focused ultrasound needs to be further studied. BBB opening induced by FUS is associated with the risk of cerebral hemorrhage and edema, and the physiological function changes of patients need to be monitored to minimize adverse reactions. All in all, the etiology of AD is complex, and how to choose the therapeutic target determines how to choose the focused ultrasound irradiation mode, which still needs further exploration and research. In the future, a large number of clinical studies are still needed to address the above questions to clarify the safety and effectiveness of FUS, so as to bring greater benefits and hope for the treatment of AD.

## Author contributions

XM: Writing – original draft, Writing – review & editing. TL: Methodology, Writing – original draft, Writing – review & editing. LD: Validation, Writing – original draft, Writing – review & editing. TH: Methodology, Writing – original draft, Writing – review & editing.

## References

[1] Karran E De Strooper B. The amyloid cascade hypothesis: are we poised for success or failure? J Neurochem. (2016) 139:237–52. doi: 10.1111/jnc.13632 27255958

[2] Usenovic M Niroomand S Drolet RE Yao L Gaspar RC Hatcher NG. Internalized tau oligomers cause neurodegeneration by inducing accumulation of pathogenic tau in human neurons derived from induced pluripotent stem cells. J Neurosci. (2015) 35:14234–50. doi: 10.1523/JNEUROSCI.1523-15.2015, PMID: 26490863 PMC6605424

[3] Ferreira ST Lourenco MV Oliveira MM De Felice FG. Soluble amyloid-β oligomers as synaptotoxins leading to cognitive impairment in Alzheimer’s disease. Front Cell Neurosci. (2015) 9:191. doi: 10.3389/fncel.2015.00191 26074767 PMC4443025

[4] Arriagada PV Growdon JH Hedley-Whyte ET Hyman BT. Neurofibrillary tangles but not senile plaques parallel duration and severity of Alzheimer’s disease. Neurology. (1992) 42:631–9. doi: 10.1212/WNL.42.3.631 1549228

[5] Grothe MJ Schuster C Bauer F Heinsen H Prudlo J Teipel SJ. Atrophy of the cholinergic basal forebrain in dementia with Lewy bodies and Alzheimer’s disease dementia. J Neurol. (2014) 261:939–48. doi: 10.1007/s00415-014-7439-z 25059393

[6] Hynynen K Jolesz FA. Demonstration of potential noninvasive ultrasound brain therapy through an intact skull. Ultrasound Med Biol. (1998) 24:275–83. doi: 10.1016/S0301-5629(97)00269-X, PMID: 9550186

[7] Leinenga G Langton C Nisbet R Gotz J. Ultrasound treatment of neurological diseases — current and emerging applications. Nat Rev Neurol. (2016) 12:161–74. doi: 10.1038/nrneurol.2016.13 26891768

[8] Krishna V Sammartino F Rezai A. A review of the current therapies, challenges, and future directions of transcranial focused ultrasound technology: advances in diagnosis and treatment. JAMA Neurol. 75:246–54. doi: 10.1001/jamaneurol.2017.3129 29228074

[9] Dubey S. Clinically approved IVIg delivered to the hippocampus with focused ultrasound promotes neurogenesis in a model of Alzheimer’s disease. Proc Natl Acad Sci U S A. (2020) 117:32691–700. doi: 10.1073/pnas.1908658117, PMID: 33288687 PMC7768694

[10] Eguchi K. Whole-brain low-intensity pulsed ultrasound therapy markedly improves cognitive dysfunctions in mouse models of dementia – crucial roles of endothelial nitric oxide synthase. Brain Stiniul. (2018) 11:959–73. doi: 10.1016/j.brs.2018.05.012, PMID: 29857968

[11] Ibsen S Tong A Schutt C Esener S Chalasani SH. Sonogenetics is a non-invasive approach to activating neurons in *Caenorhabditis elegans*. Nat Commun. (2015) 6:8264. doi: 10.1038/ncomms9264, PMID: 26372413 PMC4571289

[12] Lipsman N Meng Y Bethune AJ Huang Y Lam B Masellis M. Blood-brain barrier opening in Alzheimer's disease using MR-guided focused ultrasound. Nat Commun. (2018) 9:2336. doi: 10.1038/s41467-018-04529-6, PMID: 30046032 PMC6060168

[13] Xhima K. Focused ultrasound delivery of a selective TrkA agonist rescues cholinergic function in a mouse model of Alzheimer's disease. Sci. Adv. (2020) 6:eaax6646. doi: 10.1126/sciadv.aax6646 32010781 PMC6976301

[14] Nisbet RM. Combined effects of scanning ultrasound and a tau-specific single chain antibody in a tau transgenic mouse model. Brain. (2017) 140:1220–30. doi: 10.1093/brain/awx052, PMID: 28379300 PMC5405237

[15] Pandit R Leinenga G Götz J. Repeated ultrasound treatment of tau transgenic mice clears neuronal tau by autophagy and improves behavioral functions. Theranostics. (2019) 9:3754–67. doi: 10.7150/thno.34388, PMID: 31281511 PMC6587352

[16] Raymond SB Treat LH Dewey JD McDannold NJ Hynynen K Bacskai BJ. Ultrasound enhanced delivery of molecular imaging and therapeutic agents in Alzheimer’s disease mouse models. PLoS One. (2008) 3:e2175. doi: 10.1371/journal.pone.0002175, PMID: 18478109 PMC2364662

[17] Jordão JF Thévenot E Markham-Coultes K Scarcelli T Weng YQ Xhima K. Amyloid-β plaque reduction, endogenous antibody delivery and glial activation by brain-targeted, transcranial focused ultrasound. Exp Neurol. (2013) 248:16–29. doi: 10.1016/j.expneurol.2013.05.008, PMID: 23707300 PMC4000699

[18] Stefani A. Neurotrophins as therapeutic agents for Parkinson's disease; new chances from focused ultrasound? Front Neurosci. (2022) 16:846681. doi: 10.3389/fnins.2022.846681, PMID: 35401084 PMC8990810

[19] Lee EJ Fomenko A Lozano AM. Magnetic resonance guided focused ultrasound: current status and future perspectives in thermal ablation and blood-brain barrier opening. J Korean Neurosurg Soc. (2019) 62:10–26. doi: 10.3340/jkns.2018.0180, PMID: 30630292 PMC6328789

[20] Mehta RI. Blood-brain barrier opening with MRI-guided focused ultrasound elicits meningeal venous permeability in humans with early Alzheimer disease. Radiology. (2021) 298:654–62. doi: 10.1148/radiol.2021200643, PMID: 33399511 PMC7924515

[21] Hynynen K McDannold N Vykhodtseva N Jolesz FA. Noninvasive MR imaging-guided focal opening of the blood-brain barrier in rabbits. Radiology. (2001) 220:640–6. doi: 10.1148/radiol.2202001804 11526261

[22] Leinenga G Koh WK Golz J. Scanning ultrasound in the absence of blood-brain barrier opening is not sufficient to clear beta-amyloid plaques in the APP23 mouse model of Alzheimer's disease. Brain Res Bull. (2019, 2019) 68:2499–508. doi: 10.1016/j.brainresbull.2019.08.002 31400496

[23] Karakatsani ME. Unilateral focused ultrasound-induced blood brain barrier opening reduces phosphorylated tau from the rTg4510 mouse model. Theranostics. (2019) 9:5396–411. doi: 10.7150/thno.28717, PMID: 31410223 PMC6691580

[24] Lee Y. Improvement of glymphatic-lymphatic drainage of beta-amyloid by focused ultrasound in Alzheimer's disease model. Sci Rep. (2020) 10:16144. doi: 10.1038/s41598-020-73151-8, PMID: 32999351 PMC7527457

[25] Shen Y. Ultrasound with microbubbles improves memory, ameliorates pathology and modulates hippocampal proteomic changes in a triple transgenic mouse model of Alzheimer's disease. Theranostics. (2020) 10:11794–819. doi: 10.7150/thno.44152, PMID: 33052247 PMC7546002

[26] Mestre H Kostrikov S Mehta RI Nedergaard M. Perivascular spaces, glymphatic dysfunction, and small vessel disease. Clin Sci (Lond). (2017) 131:2257–74. doi: 10.1042/CS20160381, PMID: 28798076 PMC5567781

[27] Jordão JF Ayalagrosso CA Markham K Huang Y Chopra R McLaurin J. Antibodies targeted to the brain with image-guided focused ultrasound reduces amyloid-β plaque load in the TgCRND8 mouse model of Alzheimer's disease. PLoS One. (2010) 5:e10549. doi: 10.1371/journal.pone.0010549, PMID: 20485502 PMC2868024

[28] Burgess A Dubey S Yeung S Hough O Eterman N Aubert I. Alzheimer disease in a mouse model: MR imaging-guided focused ultrasound targeted to the hippocampus opens the blood-brain barrier and improves pathologic abnormalities and behavior. Radiology. (2014) 273:736–45. doi: 10.1148/radiol.14140245, PMID: 25222068 PMC4314115

[29] Poon CT Shah K Lin CT Tse R Kim KK Mooney S. Time course of focused ultrasound effects on β-amyloid plaque pathology in the TgCRND8 mouse model of Alzheimer’s disease. Sci Rep. (2018) 8:14061. doi: 10.1038/s41598-018-32250-3, PMID: 30232364 PMC6145880

[30] Leinenga G Götz J. Scanning ultrasound removes amyloid-β and restores memory in an Alzheimer’s disease mouse model. Sci Transl Med. (2015) 7:278ra33. doi: 10.1126/scitranslmed.aaa2512 25761889

[31] Li L Xu B Zhu Y Chen L Sokabe M Chen L. DHEA prevents Aβ25-35-impaired survival of newborn neurons in the dentate gyrus through a modulation of PI3K-Akt-mTOR signaling. Neuropharmacology. (2010) 59:323–33. doi: 10.1016/j.neuropharm.2010.02.009, PMID: 20167228

[32] Jalali S Huang Y Dumont DJ Hynynen K. Focused ultrasound-mediated bbb disruption is associated with an increase in activation of AKT: experimental study in rats. BMC Neurol. (2010) 10:114. doi: 10.1186/1471-2377-10-114, PMID: 21078165 PMC3020671

[33] Chen KT Wei KC Liu HL. Theranostic strategy of focused ultrasound induced blood-brain barrier opening for CNS disease treatment. Front Pharmacol. (2019) 10:86. doi: 10.3389/fphar.2019.00086, PMID: 30792657 PMC6374338

